# Efficacy Comparison of Pulsed Dye Laser vs. Microsecond 1064-nm Neodymium:Yttrium-Aluminum-Garnet Laser in the Treatment of Rosacea: A Meta-Analysis

**DOI:** 10.3389/fmed.2021.798294

**Published:** 2022-01-20

**Authors:** Yuanchao Li, Rupeng Wang

**Affiliations:** Department of Dermatology, Rheumatology, and Immunology, Xinqiao Hospital of Army Medical University, Chongqing, China

**Keywords:** rosacea, pulsed dye laser, Nd:YAG, meta-analysis, efficacy

## Abstract

**Purpose:**

The advantage of pulsed dye laser (PDL) for the treatment of rosacea is not yet clear. This meta-analysis compared the curative effect of PDL to neodymium:yttrium-aluminum-garnet (Nd:YAG) laser for the treatment of rosacea.

**Methods:**

The PubMed, Embase, and Cochrane Library databases were searched for clinical studies on the efficacy of PDL for the treatment of rosacea through October 13, 2021, and heterogeneity tests among studies were evaluated. Meta-analysis was conducted to combine the effects of physicians' clinical assessments, patient global assessment, erythema index, and visual analog scale.

**Results:**

A total of 326 articles were obtained from three databases and ten articles were finally included. The clinical improvements of >50% clearance of up to 68.6% in the PDL group and 71.4% in the control group, and the subjective satisfaction rate of patients in the PDL group of 88.6% compared to 91.4% in the Nd:YAG group, but there were no significant differences in the rates of patients with rosacea with clinical improvement (>50% clearance) (relative risk [RR] = 0.94, 95% confidence interval [CI]: 0.75–1.17, *P* = 0.578) or patient subjective satisfaction rate (RR = 0.96, 95% CI: 0.70–1.33, *P* = 0.808) between PDL and Nd:YAG groups for rosacea treatment. Also, the pain score for PDL and Nd:YAG were not significant (mean = 3.07, 95% CI: 1.82–4.32, *P* = 0.115).

**Conclusion:**

Two treatments all showed clinical efficacy and patient satisfaction for the treatment of rosacea, with no significant differences observed between treatments. The pain scores for PDL and Nd:YAG were not significant.

## Highlights

- Two methods all showed clinical efficacy and satisfaction for the therapy of rosacea.
- Clinical efficacy and satisfaction were similar between the two treatments.
- The pain scores for PDL and Nd:YAG were not significant.

## Introduction

Rosacea, also known as rose acne, is a facial inflammatory disease with clinical manifestations of transient or persistent erythema, papules, pustules, burning sensation, pruritus, or telangiectasia in the middle of the face (1). It is well known that rosacea is related to genetic factors, congenital, and adaptive immune system disorders, vascular, and neurological dysfunction, *Demodex folliculorum*, inflammation, and microorganism factors (2, 3), while the specific pathogenesis of rosacea is not fully understood. A study conducted in a genome-wide association identified two single nucleotide polymorphisms in European individuals with rosacea which might predispose them to the development of rosacea (4). Immune dysregulation is also an important cause of rosacea. In patients with rosacea, the innate immune system was activated and thus increased cytokines and antimicrobial peptides (5). Increased human cationic antibacterial protein 18kDa (hCAP18) is cleaved into LL-37, which promotes angiogenesis, induces leukocyte chemotaxis, and is involved in the production of proinflammatory cytokines (6). Activation of transient receptor potential (TRPs) in patients with rosacea leads to the release of mediators of neurogenic inflammation and pain, such as substance P and calcitonin gene-related peptide. These vasoregulatory neuropeptides are critical mediators that induce sustained flushing that is a characteristic of rosacea (5). The reported aggravating factors for rosacea include high temperature, alcohol, sunlight, stress, menstruation, drugs, and certain foods (7). The onset age of rosacea is usually older than that for acne, and rosacea mainly occurs in middle-aged people.

Clinically, the overall incidence of rosacea is increasing (8). Rosacea not only affects the normal life of patients, but may also progress to capacitive skin diseases and blindness (6, 9). The diversity of clinical manifestations of rosacea requires a variety of appropriate and targeted treatment methods, including skincare, local or systemic drug treatment, or physical and surgery therapies (10, 11). However, telangiectatic (ET) and rhinophyma (RP) changes have limited therapeutic effects on drugs, and traditional methods (12).

Recently, pulsed dye laser (PDL) has been widely used to treat rosacea-related erythema and telangiectasia (13, 14). PDL emits pulsed laser energy at wavelengths of 585 or 595 nm, which is absorbed by oxyhemoglobin to inhibit endothelial cell formation, reduce angiogenesis, and destruct the existing vessels (15). Many studies have also demonstrated the efficacy of other photoelectric treatments including long-pulsed neodymium: yttrium-aluminum-garnet laser (Nd:YAG) on rosacea (16). Heterogeneous therapeutic effect for rosacea occurred in different studies in which PDL reduced more facial redness than Nd:YAG in the study of Alam (17) while exhibited no significant differential treatment outcome in the study of Hyun-Min (18). The various results might stem from the sample size, study method like fluence, and so on. Meta-analysis provides a well-done understanding for combining the different studies with differential features. A systematical meta-analysis compared the differences between the two treatments and showed that the PDL leads to worse improvement for erythema (19), providing a general understanding for researchers. However, the study was conducted in March 2020, with only 5 papers included, and did not perform the methodological quality and heterogeneity test. The meta-analysis which evaluated the differences between PDL and Nd: YAG for the treatment of rosacea in terms of curative effect and patient satisfaction needed to be further conducted to provide evidence for clinical treatment.

## Methods

### Literature Retrieval Strategy

Systematic literature retrieval was performed from the PubMed, Embase, Cochrane Library databases according to the pre-defined retrieval strategy. The search keywords were Rosacea AND [PDL OR (Pulsed Dye Laser)] OR [Nd:YAG OR (neodymium: yttrium aluminum-garnet laser)]. Subject and free words were performed in this search, and the search format changed according to the characteristics of the database (for the specific different retrieval steps of each database, see Supplementary Tables 1–3). The retrieval time was up to October 13, 2021, and there was no language restriction. In addition, we also manually retrieved the paper articles, and screened the bibliographies of relevant systematic reviews for more sources that could be included in the meta-analysis.

### Inclusion and Exclusion Criteria

The inclusion criteria were (1) subjects with erythemato-telangiectatic rosacea or papulopustular rosacea patients; (2) the studies reported that the efficacy or safety of PDL vs. Nd:YAG treated rosacea or the efficacy or safety of PDL/Nd:YAG vs. other treatment treated rosacea, or the single-arm study of PDL/Nd:YAG treated rosacea; (3) study outcomes, included clinical efficacy, subjective improvement, erythema index, or pain score (visual analog scale), after the intervention; and (4) study types were randomized controlled trial (RCT) or prospective cohort studies.

The exclusion criteria were (1) studies using PDL in combination with other drugs or interventions; (2) studies with fewer than 10 patients to reduce the risk of publication bias; (3) studies with unclear rosacea type; (4) reviews, letters, comments, etc.; and (5) repeated publications or use of the same data for multiple articles (only the study with the most complete information was included, and the rest were excluded).

### Data Extraction and Quality Evaluation

Two investigators independently completed the literature screening according to the inclusion and exclusion criteria and independently carried out data extraction according to the standardized table designed in advance. The extracted information included the first author name and article publication year, the area where the study was carried out, participant information (age, sample size, and sex), intervention plans, and outcome indicators. After data extraction, two investigators exchanged audit extraction forms. Any inconsistencies were resolved by discussion.

The Cochrane Collaboration's tool for evaluating risk was used to evaluate the quality of the included RCT studies (20). In addition, the Newcastle-Ottawa scale (NOS) was conducted to assess the methodological quality of the cohort studies (21).

Differences in the quality evaluations were resolved by reaching a consensus after a group discussion with the third author.

### Statistic Analysis

There were two comparisons in this meta-analysis, including (1) the efficacy or safety of PDL, the Nd:YAG treated rosacea were respectively obtained, and the Mean [95% confidence intervals (CIs)] was used to merge continuous variables, and the Incidence Rate (IR) and 95%CI were utilized to merge classified variable. The combined results between two groups were performed the indirect comparison, and the Altman, DG (24), and Delong, ER (25) methods were used to conduct the difference significance test; (2) direct comparison: the efficacy or safety of PDL vs. Nd:YAG treated rosacea were obtained, weighted mean differences (WMDs), and their 95% confidence intervals (CIs) were used to merge continuous variables, while relative risks (RRs) with 95% CIs were used to the merge effect values of classified variables. Cochran's Q and I^2^ tests were applied for heterogeneity assessment (26). If significant heterogeneity among studies was evaluated, defined as Q Statistic P ≤ 0.05 or I^2^ > 50%. The random-effects model was conducted on the meta-analysis because there was a large clinical and methodological heterogeneity between the included studies. All statistical analyses in this study were performed using RevMan5.3 (Copenhagen: The Nordic Cochrane Center and The Cochrane Collaboration) and Stata15.0 (Stata Corp., TX, USA) software.

## Results

### Literature Retrieval

**Figure 1:**
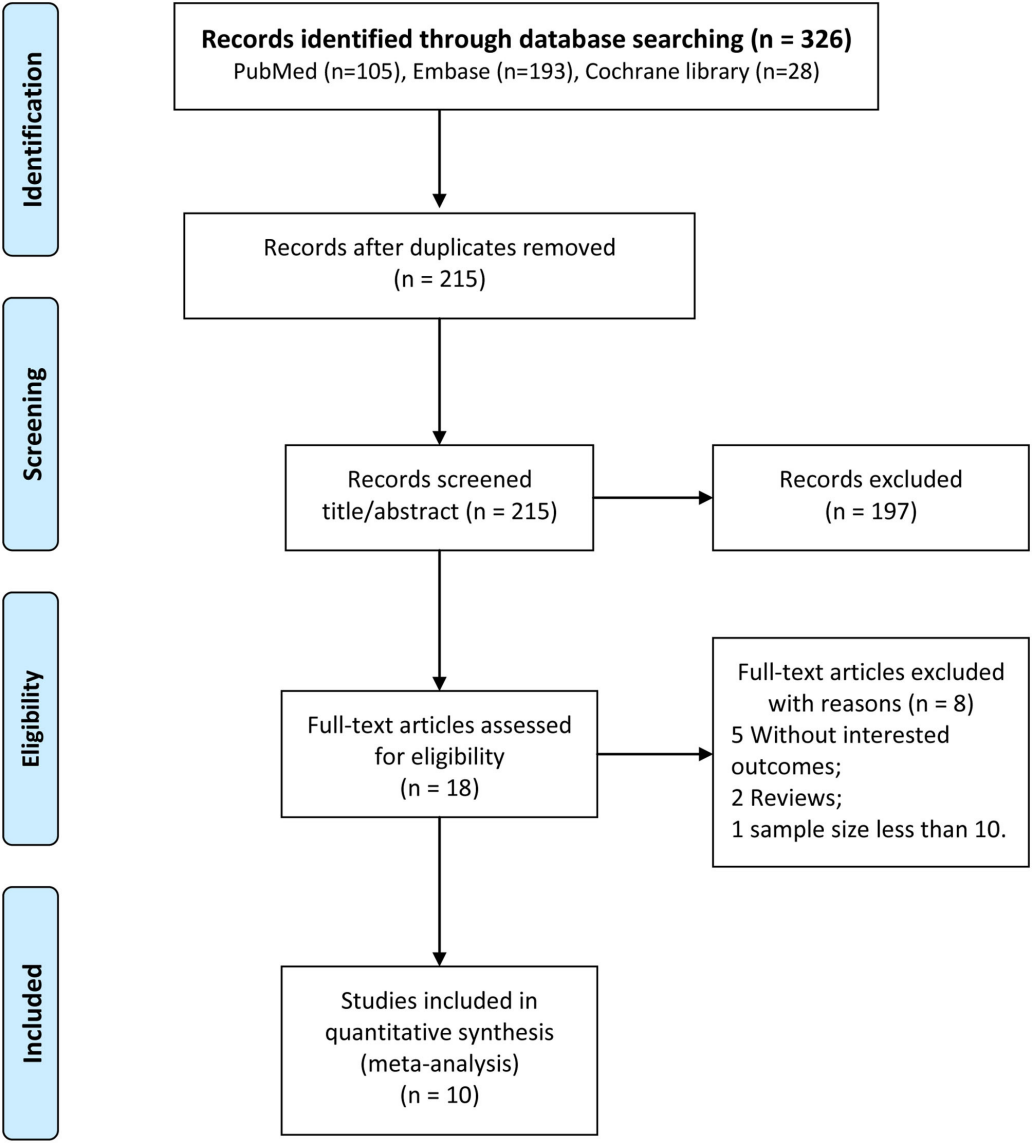
Flowchart of study retrieval.

The process of literature screening is shown in Figure 1. A total of 105, 193, and 28 articles were initially identified in the PubMed, Embase, and Cochrane Library databases, respectively. After excluding repeated articles, 215 articles remained,197 of which did not meet the inclusion criteria after browsing the titles and abstracts. Eight of the remaining 18 articles were excluded after reading the full texts. No additional eligible articles were found through manual searching; finally, ten articles (16–18, 22, 23, 27–31) were included in this study.

### Characteristics of the Included Studies

The included papers were published in 2011–2020, and distributed in the United States, Egypt, and South Korea. Among the ten included studies, six studies were RCTs (17, 23, 27, 28, 30, 31) and four studies were prospective cohorts (17, 25, 15, 28). The total sample size of the six studies was 235 cases. There was no significant difference in age between the PDL and control groups in each study; however, differences in Fitzpatrick skin types were observed among participants of each study. Other baseline information is detailed in Table 1.

**Table 1:** Characteristics of ten studies included in this meta-analysis.

**Study**	**Area**	**Design, Control**	**Intervention**	**Age, years**	**Type of rosacea**	**Fitzpatrick skin types**	**Group**	***n*, M/F**	**Outcomes**	**Devices setting**
Alam et al. (17)	USA	RCT, split-face	Received 4 treatments each at one month intervals.	42	ETR	1 I, 11 II, 2 III	PDL	14, 8/6	VAS	595 nm(non-purpuragenic), fluence, 7.5 J/cm^2^; spot size, 10 mm; pulse duration, 6 ms; DCD, 30 ms/20 ms.
							Nd:YAG	14, 8/6		1064 nm, fluence, 6 J/cm^2^; spot size, 8 mm; pulse duration, 0.3 ms.
Bulbul Baskan and Akin Belli (29)	Turkey	No-RCT, no control	Varied from one to four sessions with 4–6-week intervals.	45.36 ± 8.93	13 ETR, 1 PPR	2 I, 11 II, 1 III	PDL	14, 5/9	PCA, PGA	595 nm (non-purpuragenic), spot of 7–10 mm, fluence of 8–12 J/cm^2^, and median pulse duration of 10–20 ms.
Campos et al. (30)	Portugal	RCT, split-face	The sessions were applied with 3–4-week intervals.	52.9 ± 15.9	ETR	NR	PDL	27, 10/17	VAS	595 nm (purpuragenic), fluence of 6.0 J/cm^2^, spot size of 7 mm, pulse duration of 0.5 ms, DCD level 3 of 5.
Kim et al. (23)	Korea	RCT, split-face	Three sessions at 4-week intervals.	43.4 (35–69)	20 ETR, 10 PPR	NR	PDL	30, 11/19	PCA, PGA	595 nm (subpurpuragenic), 7 mm spot, fluence 8–9 J/cm^2^, pulse duration of 6 ms, and DCD setting of 30 ms.
Kim et al. (27)	Korea	RCT, split-face	Three times at 3-week intervals.	43 (28–67)	13 ETR, 2 PPR	2 III, 10 IV, 3 V	PDL	15, 4/11	PCA	585 nm (subpurpuragenic), fluence 7–9 J/cm^2^, 10 ms pulse duration, 7 mm spot size.
Kwon et al. (16)	Korea	No-RCT, split-face	Three times with 4-week intervals.	55.6 (34–75)	ETR	17 III, 3 IV	PDL	20, 12/8	PCA, PGA, AE	595 nm (non-purpuragenic), 7 mm spot size, 6 ms pulse duration, fluence 9J, 30/20 ms DCD.
							Nd:YAG	20, 12/8		1064 nm, 2 mm spot size, 10–25 ms pulse durations, 150–250 J/cm^2^.
Osman et al. (31)	Egypt	RCT, person-by-person	Four sessions with 4-week intervals.	38.07 ± 9.11	9 ETR, 6 PPR	12 III, 3 IV	PDL	15, 3/12	PGA	595 nm (subpurpuragenic), 5 to 7 mm spot size, a duration of 450 microsecond, a fluence ranging from 5 to 6.5 J/cm^2^.
Salem et al. (18)	Egypt	No-RCT, split-face	Three sessions, 4 weeks apart.	43.5 ± 8.7	ETR	15 III	PDL	15, 0/15	PCA, PGA	595 nm (subpurpuragenic), fluence 12 J/cm^2^, spot size 7 mm, and pulse duration 6 ms, with 10% overlap of treatment spots.
							Nd:YAG	15, 0/15		1064 nm, fluence 22 J/cm^2^, spot size 18 mm, and pulse duration 10 ms, with non-overlapping pulses.
Ekin Mese Say and Gökdemir (22)	Turkey	No-RCT, no control	The sessions were applied with 3–4-week intervals.	47.05 (24–78)	ETR, PPR	42 II, 24 III	Nd:YAG	66, 22/44	PCA, PGA	1064 nm, 2 to 3 mm spot size, 100 to 160 J/cm^2^, 15 to 20 ms pulse durations.
					PPR	16 II, 11 III	Nd:YAG	27, 12/15		
					ETR	26 II, 13 III	Nd:YAG	39, 10/29		
Kim et al. 2016 (23)	Korea	RCT, person-by-person	Four treatments with monthly intervals.	48.6 ± 11.3	ETR, PPR	III-V	PDL	19, 11/8	PCA, PGA	585 nm (non-purpuragenic), fluence 7 J/cm^2^, spot size 10 mm, pulse width of 6 ms.

*M, male; F, female; RCT, randomized controlled trial; DCD, dynamic cooling device; ms, milliseconds; PDL, pulsed dye laser; Nd:YAG, neodymium:yttriumaluminum-garnet laser; IPL, intense pulsed light; IGA, physicians' clinical assessment; PGA, patient global assessment; VAS, visual analog scale; ETR, erythemato-telangiectatic rosacea; PPR, papulopustular rosacea; NR, not reported; AE, adverse events; VAS, visual analog scale*.

### Quality of the Included Studies

**Figure 2:**
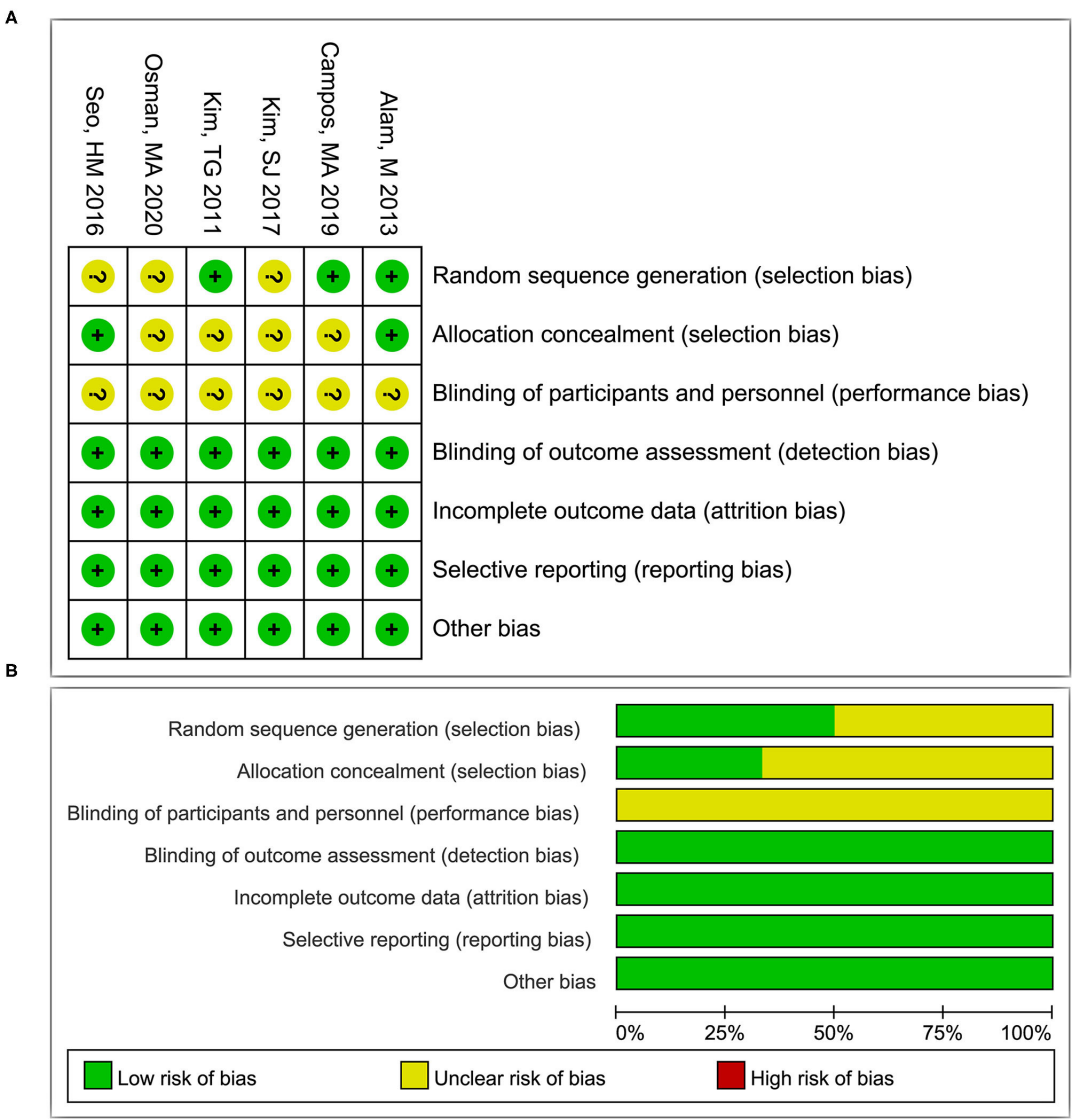
The risk of bias assessments in each item for studies **(A)** and summary of the risk of bias in each item by all studies **(B)**. +, low risk of bias; -, high risk of bias; ?, unclear risk of bias.

The methodology quality of each included study was evaluated. Although six RCT studies implemented the blinding of subjects, the clinicians were not blinded; thus the evaluations of the “Blinding of participants and personnel” for all three included studies were “unclear risk”. Only Alam et al. (17), Campos et al. (30), and Kim et al. (27) reported specific randomization, while the studies by Alam et al. (17) and Seo et al. (23)reported allocation concealment. All three studies showed low risks in terms of “detection bias”, “attrition bias”, “reporting bias”, and “other bias”. Overall, the methodology quality of the included RCTs was moderate (Figure 2). The results of the NOS evaluation of the four prospective cohort studies showed moderate-high quality (Table 2).

**Table 2:** Quality assessment of the prospective cohort studies with Newcastle-Ottawa quality assessment scale.

**Study**	**Representati-veness of the exposed cohort**	**Selection of the unexposed cohort**	**Ascertainment of exposure**	**Outcome of interest not present at start of study**	**Control for important factor or additional factor**	**Outcome assessment**	**Follow-up long enough for outcomes to occur**	**Adequacy of follow-up of cohorts**	**Total quality scores**
Bulbul Baskan and Akin Belli (29)	☆	–	☆	☆	–	☆	☆	☆	6
Kwon et al. (16)	–	☆	☆	☆	☆	☆	☆	☆	7
Salem et al. (18)	☆	☆	☆	☆	☆	☆	☆	☆	8
Ekin Mese Say and Gökdemir (22)	☆	–	☆	☆	–	☆	☆	☆	6

### Results of Meta-Analysis

**Figure 3:**
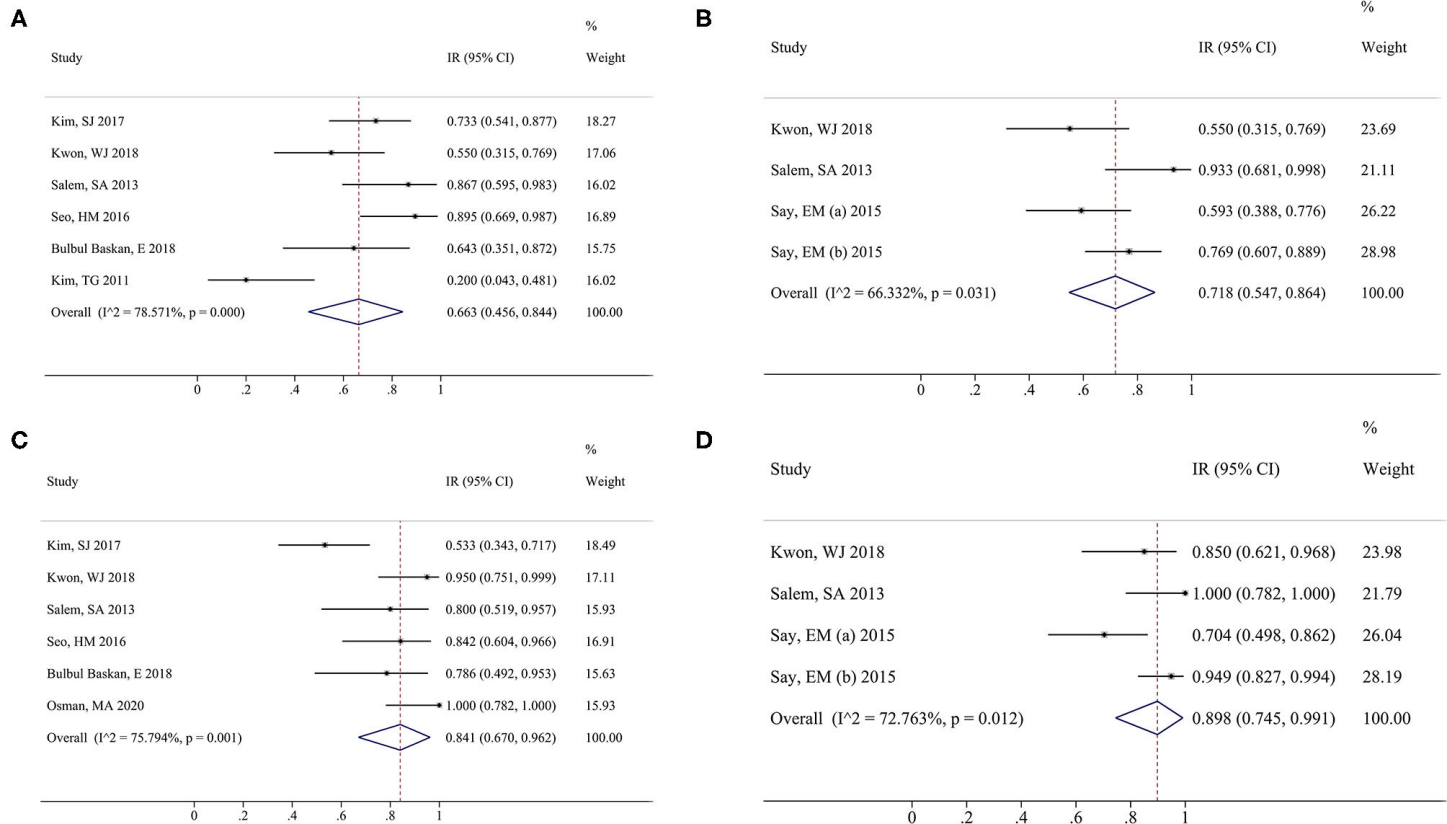
Forest plot of overall risk ratios of physicians' clinical assessment (PCA) and patient global assessment (PGA) in the pulsed dye laser (PDL) group vs. neodymium:yttrium-aluminum-garnet (Nd:YAG) groups. PCA of PDL group **(A)** and Nd:YAG group **(B)**; PGA of PDL group **(C)** and Nd:YAG group **(D)**. Squares indicate the estimates for the corresponding study; the size of the square is proportional to the weight of the study to the overall estimate. Diamonds indicate an overall pooled estimate while horizontal lines represent the 95% CI. PDL, pulsed dye laser; Nd:YAG, neodymium:yttrium-aluminum-garnet.

The main outcomes and results of each of the ten included articles were listed in Table 3. A total of six studies (16, 18, 23, 27–29) reported the physicians' clinical assessment (PCA) of the PDL group, and the combined results showed IR (95%CI) = 0.663 (0.456–0.844), and significant heterogeneity was found between these studies (I^2^ = 78.6%, *P* < 0.001) (Figure 3A). Three studies (17, 25, 15) reported the PCA of Nd:YAG and the combined results showed IR (95%CI) = 0.718 (0.547–0.864), and significant heterogeneity was found between these studies (I^2^ = 66.3%, *P* = 0.031) (Figure 3B). No significant difference between these two groups was detected in the combined results (*P* = 0.667).

**Table 3:** The main outcomes and results of each of the included study.

**Study**	**Area**	**Type of rosacea**	**Group**	***n***	**Outcome**	**Main results**
Alam et al. (17)	USA	ETR	PDL vs. Nd:YAG	14/14	VAS	Pain varied with Nd:YAG associated with less pain, at 3.07 (0.64), than PDL at 3.87 (0.65)
Bulbul Baskan and Akin Belli (29)	Turkey	13 ETR, 1 PPR	PDL	14	PCA	9 patients with clinical improvement
					PGA	11 patients with subjective satisfacted
Campos et al. (30)	Portugal	ETR	PDL	27	VAS	5.33 ± 2.9 after the third session
Kim et al. (23)	Korea	20 ETR, 10 PPR	PDL	30	PCA	22 patients with clinical improvement
					PGA	16 patients with subjective satisfacted
Kim et al. (27)	Korea	13 ETR, 2 PPR	PDL	15	PCA	3 patients with clinical improvement
Kwon et al. (16)	Korea	ETR	PDL vs. Nd:YAG	20/20	PCA	Improvement with 11 caes in Nd-YAG and 11 cases in PDL
					PGA	Satisfacted with 19 cases in Nd-YAG and 17 cases in PDL
					AE	Only Purpura with significant difference, 18 (PDL) vs. 3 cases (Nd-YAG); Edema, Erythema, Hyperpigmentation, or Vesicles, was no significant difference.
Osman et al. (31)	Egypt	9 ETR, 6 PPR	PDL	15	PGA	15 patients with subjective satisfacted
Salem et al. (18)	Egypt	ETR	PDL vs. Nd:YAG	15/15	PCA	Improvement with 14 caes in Nd-YAG and 13 cases in PDL
					PGA	Satisfacted with 15 cases in Nd-YAG and 12 cases in PDL
Ekin Mese Say and Gökdemir (22)	Turkey	PPR	Nd:YAG	27	PCA	16 patients with clinical improvement
					PGA	19 patients with subjective satisfacted
		ETR	Nd:YAG	39	PCA	30 patients with clinical improvement
					PGA	37 patients with subjective satisfacted
Kim et al. (23)	Korea	ETR, PPR	PDL	19	PCA	17 patients with clinical improvement
					PGA	16 patients with subjective satisfacted

*AE, adverse events; ETR, erythemato-telangiectatic rosacea; Nd:YAG, neodymium:yttriumaluminum-garnet laser; PCA, physicians' clinical assessment; PDL, pulsed dye laser; PGA, patient global assessment; PPR, papulopustular rosacea*.

In addition, a total of six studies (16, 18, 23, 28, 29, 31) reported the patient global assessment (PGA) of the PDL group, and the combined results showed IR (95%CI) = 0.841 (0.670–0.962), and significant heterogeneity was found between these studies (I^2^ = 75.8%, *P* = 0.001) (Figure 3C). Three studies (17, 25, 15) reported the PGA of Nd:YAG and the combined results showed IR (95%CI) = 0.898 (0.745–0.991), and significant heterogeneity was found between these studies (I^2^ = 72.8%, *P* = 0.012) (Figure 3D). There was no significant difference between the two groups (*P* = 0.559).

**Figure 4:**
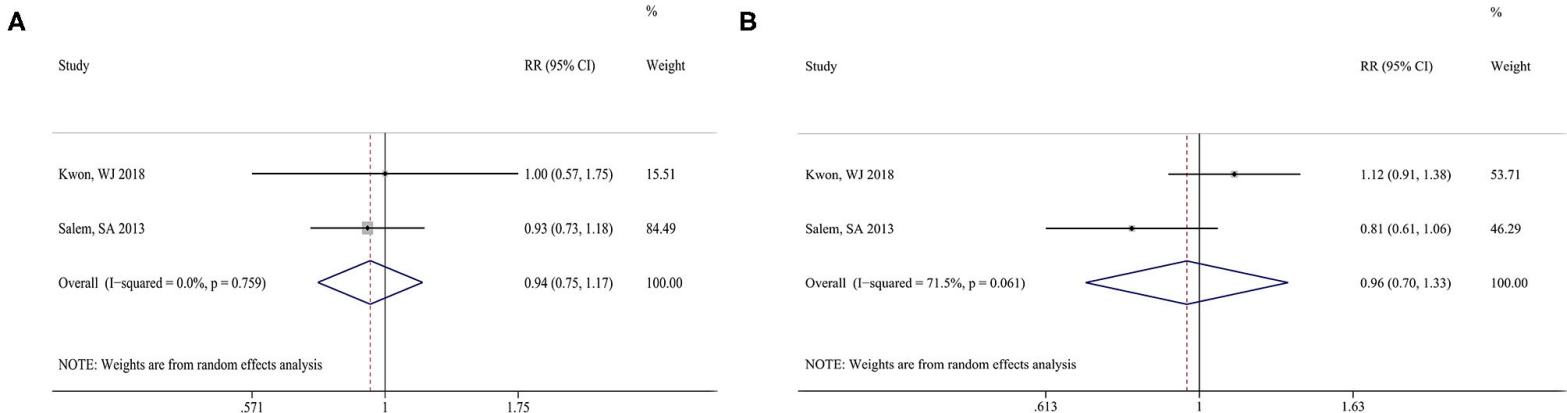
Forest plot of the overall risk ratios of clinical efficacies of rosacea treated compared with PDL than Nd:YAG. PCA **(A)** and PGA **(B)**. PDL, pulsed dye laser; Nd:YAG, neodymium:yttrium-aluminum-garnet.

The clinical efficacies of PDL and Nd:YAG were compared based on PCA to evaluate clinical improvement (defined as of >50% clearance, a subjective method for blinded dermatologists to assess the improvement in the severity of erythema). A total of two articles (17, 15) reported this outcome, and no significant heterogeneity was found between the two studies (I^2^ = 0.0%, *P* = 0.759); thus, a fixed-effect model was used to combine data. The result showed clinical improvements of >50% clearance of up to 68.6% in the PDL group and 71.4% in the Nd:YAG group, but no significant differences in the rates of patients with rosacea with clinical improvement between the two groups (RR = 0.94, 95%CI: 0.75–1.17, *P* = 0.578) (Figure 4A).

Two studies (17, 15) reported the results of PGA in the PDL and Nd:YAG groups. Significant heterogeneity was found between the two studies (I^2^ = 71.5%, *P* = 0.061). The merged result showed a subjective satisfaction rate of patients in the PDL group of 88.6% compared to 91.4% in the control group, a difference between the two groups that was not statistically significant (RR = 0.96, 95%CI: 0.70–1.33, *P* = 0.808) (Figure 4B).

**Figure 5:**
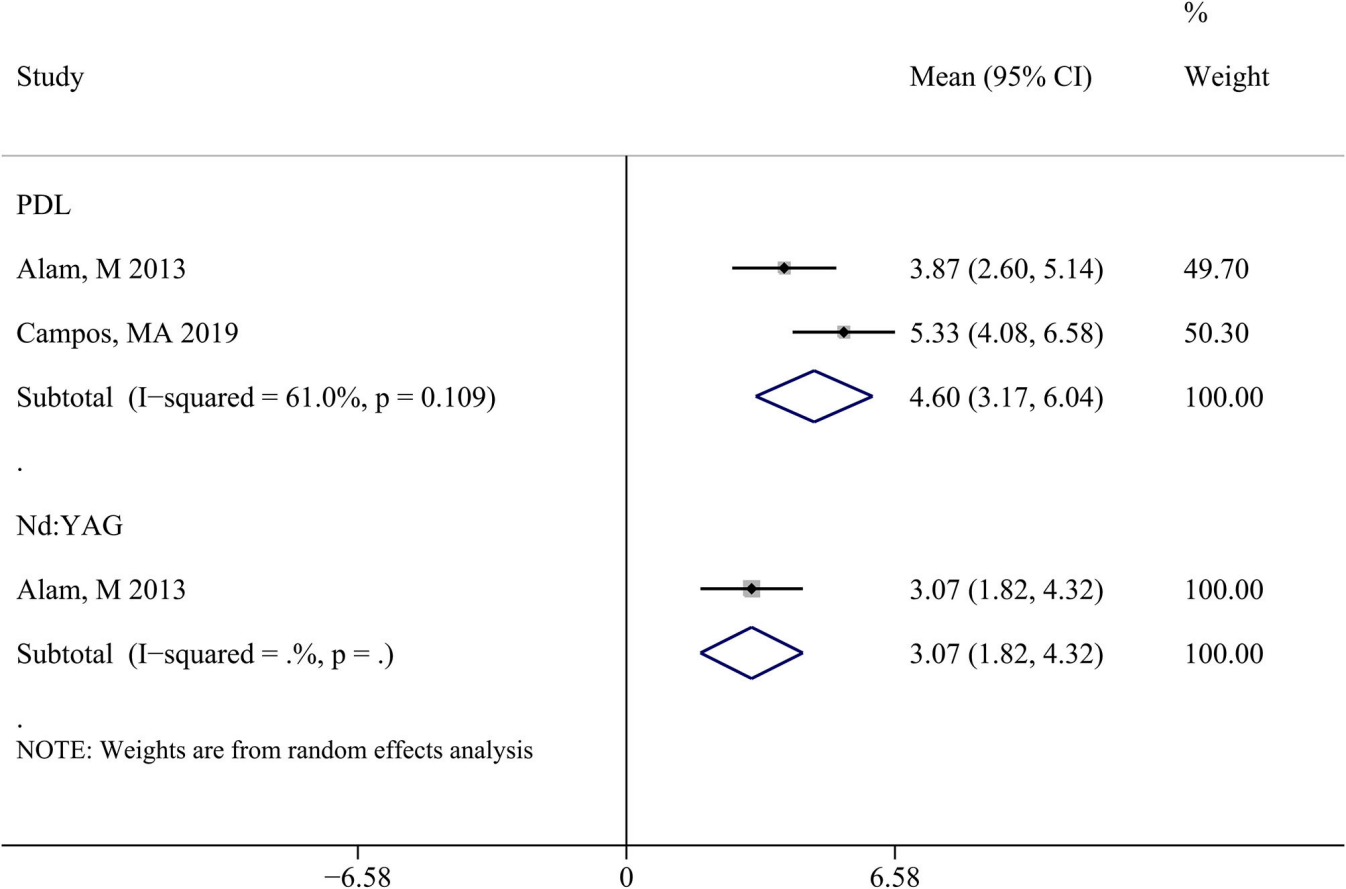
Forest plot of standard mean difference to compare pain score between PDL and Nd:YAG groups. PDL, pulsed dye laser; Nd:YAG, neodymium:yttrium-aluminum-garnet.

Two studies (17, 30) reported the pain score of the PDL group, and one study reported the pain score of the Nd:YAG group. Based on the visual analog scale (VAS) as the pain outcome index, the combined result of the PDL group showed a mean (95%CI) = 4.60 (3.17–6.04), indicating not significantly higher (*P* = 0.115) pain sensation of patients treated with PDL than that in patients treated with Nd:YAG, mean (95%CI) = 3.07 (1.82–4.32) (Figure 5).

## Discussion

To explore the different clinical effects of PDL and Nd:YAG on rosacea, PCA, PGA, VAS, and adverse events were defined as the outcome indexes. The results of our analyses showed no significant differences in the percentage of cases with clinical improvement (>50% clearance) and pain score.

Hemangiectasis in rosacea is located in deep subdermal blood vessels located approximately 3 mm deep and mainly requires long-pulse Nd:YAG laser (1064 nm) for treatment (32). Nd:YAG has a good clinical effect for the treatment of the vascular and inflammatory lesions of rosacea (22). In a double-blind RCT study comparing Nd:YAG to PDL for treating diffuse facial erythema, the efficacy of PDL was better than that of Nd:YAG, but the pain of Nd:YAG was less than that of PDL (17). Our study revealed that PDL and Nd:YAG were both effective for treating rosacea with comparable physician assessments and patient satisfaction; however, there was no statistical significance between the rosacea improvement effects of the two groups. Consistent with our findings, two experiments also point that both PDL and Nd:YAG laser have good physician assessment with no significant difference (16, 28). What is more, a meta-analysis also confirmed the treatment success throughout the physician's assessment results while no significant differences were found in the two treatment methods (19). As for the similar pain score after the treatment of PDL and Nd:YAG laser, the results were not coincident with our conclusion in two experimental studies and a meta-analysis (17, 19, 30). The reason for the disagreement might come from the study concluded in the meta-analysis being heterogenic. As for the reverse effects of the PDL and Nd:YAG laser, the main side effect of Nd:YAG was temporary erythema, without purpura, pigmentation, or scar (10). Similarly, no serious adverse events, such as pigmentation or scarring, have been reported for either PDL or Nd:YAG treatment (17, 23). These findings confirmed that the Nd:YAG laser successfully treated erythema telangiectasia rosacea with lower energy and longer pulse width without side effects. The main adverse reaction of PDL was purpura, which mainly acts on superficial and small capillaries, but can also reach deep into the dermis (30). This may partly explain why PDL feels more painful than Nd:YAG for the treatment of rosacea. An article included in the present study also reported the differential adverse events between the PDL treatment and Nd:YAG laser treatment (15), and the results showed that PDL treatment caused more purpura than Nd:YAG laser treatment. It is a pity that we cannot conduct a meta-analysis about the adverse events because only one included article reported the adverse events after treatment with PDL and Nd:YAG laser.

Our findings indicated that PDL was not superior to Nd:YAG for the treatment of rosacea, contrary to one review report (33). However, the review only referenced the redness result from Alam 2013 (17), in which redness improved by a mean of 52% after PDL treatment compared to 34% after the Nd:YAG treatment, a statistically significant difference between the methods. Although we also included Alam 2013 in our analyses, redness was not used as an assessment indicator since only this paper among all included studies reported redness data. In addition, the review ignored pain findings (33), while our results comprehensively assessed the clinical efficacy, satisfaction rates, and pain scores from several studies.

The findings in our analysis can be used to inform the selection of methods for the treatment of rosacea. However, this study had some limitations. Firstly, the numbers of included studies and sample size were both small. Secondly, the clinical and methodological heterogeneity of the included literature was relatively large. Thirdly, the examination of publication bias was limited due to the small number of included studies. The inevitable potential publication bias may have affected the robustness of the conclusions. Fourthly, it is a pity that we cannot conduct a meta-analysis about the adverse events because only one included article reported the relevant adverse events after treatment with PDL and Nd:YAG laser. More studies about the adverse events caused by treatment with PDL and/or Nd:YAG laser need to be conducted and more validation is necessary to be involved in the further meta-analysis. Finally, the exclusion criteria in this study may induce a potential applicability limitation: although the method may result in an accurate outcome of using PDL or Nd:YAD only, the absence of other therapy method limit the applicability of these results. Actually, patients often tend to select several therapy methods rather than only one therapy to improve the severity of rosacea in clinical practice.

## Conclusion

In conclusion, PDL and Nd:YAG all showed clinical efficacy and patient satisfaction for the treatment of rosacea, with no significant difference observed between treatments. Also, the analysis of the pain score between the PDL and Nd:YAG treatment exhibited no statistical significance. These findings will play an important guiding role in the clinical treatment of rosacea. However, considering the limitations of this study, such as its limited sample size, the results of this meta-analysis require verification by RCT with larger samples and higher quality.

## Data Availability Statement

The original contributions presented in the study are included in the article/Supplementary Material, further inquiries can be directed to the corresponding author/s.

## Author Contributions

YL and RW contributed to conception and design of the research and acquisition of data. YL contributed to analysis and interpretation of data, statistical analysis, and drafting the manuscript. RW contributed to the revision of the manuscript for important intellectual content. All authors have read and approved the final manuscript.

## Conflict of Interest

The authors declare that the research was conducted in the absence of any commercial or financial relationships that could be construed as a potential conflict of interest.

## Publisher's Note

All claims expressed in this article are solely those of the authors and do not necessarily represent those of their affiliated organizations, or those of the publisher, the editors and the reviewers. Any product that may be evaluated in this article, or claim that may be made by its manufacturer, is not guaranteed or endorsed by the publisher.
