# Supplementary material for: Efficacy Comparison of Pulsed Dye Laser vs. Microsecond 1064-nm Neodymium:Yttrium-Aluminum-Garnet Laser in the Treatment of Rosacea: A Meta-Analysis
Source: Front Med (Lausanne). 2022 Jan 20;8:798294. doi: 10.3389/fmed.2021.798294 (PMC8811442; doi:10.3389/fmed.2021.798294)
Supplement: Supplementary file 1 [file Data_Sheet_1.docx]

**Sup Table 1 Retrieval steps and results from PubMed (retrieval time: 20211013)**

| Search | Query | Items found |
| --- | --- | --- |
| #1 | "rosacea"[MeSH Terms] OR "rosacea"[All Fields] | 4490 |
| #2 | PDL[All Fields] OR ("lasers, dye"[MeSH Terms] OR ("lasers"[All Fields] AND "dye"[All Fields]) OR "dye lasers"[All Fields] OR ("pulsed"[All Fields] AND "dye"[All Fields] AND "laser"[All Fields]) OR "pulsed dye laser"[All Fields]) | 8817 |
| #3 | "lasers, solid state"[MeSH Terms] OR ("lasers"[All Fields] AND "solid state"[All Fields]) OR "solid-state lasers"[All Fields] OR ("neodymium"[All Fields] AND "yttrium"[All Fields] AND "aluminum"[All Fields] AND "garnet"[All Fields] AND "laser"[All Fields]) OR "neodymium yttrium aluminum garnet laser"[All Fields] | 8713 |
| #4 | #2 OR #3 | 17107 |
| #5 | #1 AND #4 | 105 |

**Sup Table 2 Retrieval steps and results from Embase**

| Search | Query | Items found |
| --- | --- | --- |
| #1 | 'rosacea'/exp OR rosacea | 7734 |
| #2 | PDL OR 'pulsed dye laser'/exp OR 'pulsed dye laser' OR (pulsed AND ('dye'/exp OR dye) AND ('laser'/exp OR laser)) | 10421 |
| #3 | ndyag OR (('neodymium'/exp OR neodymium) AND (('yttrium'/exp OR yttrium) AND 'aluminum garnet' AND ('laser'/exp OR laser)) OR “yttrium aluminum-garnet laser”) | 3360 |
| #4 | #2 OR #3 | 13662 |
| #5 | #1 AND #4 | 193 |

**Sup Table 3 Retrieval steps and results from the Cochrane Library**

| Search | Query | Items found |
| --- | --- | --- |
| #1 | MeSH descriptor: [Rosacea] in all MeSH products OR | 283 |
| #2 | (Rosacea):ti,ab,kw (Word variations have been searched) | 658 |
| #3 | #1 OR #2 | 659 |
| #4 | MeSH descriptor: [Lasers, Dye] explode all trees | 92 |
| #5 | (Pulsed Dye Laser):ti,ab,kw (Word variations have been searched) OR (PDL):ti,ab,kw (Word variations have been searched) | 675 |
| #6 | #4 OR #5 | 678 |
| #7 | ("neodymium-yttrium aluminum garnet laser"):ti,ab,kw | 141 |
| #8 | ("Nd:YAG laser"):ti,ab,kw | 1027 |
| #9 | #7 OR #8 | 1074 |
| #10 | #6 OR #9 | 1707 |
| #11 | #3 AND #10 | 29 |
| #12 | #11 in trials | 28 |
